# Does Intraoperative Ulinastatin Improve Postoperative Clinical Outcomes in Patients Undergoing Cardiac Surgery: A Meta-Analysis of Randomized Controlled Trials

**DOI:** 10.1155/2014/630835

**Published:** 2014-03-09

**Authors:** Qiu-Lan He, Fei Zhong, Fang Ye, Ming Wei, Wei-Feng Liu, Mei-Na Li, Qiao-Bo Li, Wen-Qi Huang, Lai-Bao Sun, Hai-Hua Shu

**Affiliations:** ^1^Department of Anesthesiology, The First Affiliated Hospital of Sun Yat-sen University, No. 58 Zhongshan 2nd Road, Guangzhou, Guangdong 510080, China; ^2^School of Public Health, Sun Yat-sen University, No. 74 Zhongshan 2nd Road, Guangzhou, Guangdong 510080, China; ^3^Guangzhou Municipal Center for Disease Control and Prevention, Guangzhou 0086-510080, China

## Abstract

*Introduction*. The systematic meta-analysis of randomized controlled trials (RCTs) evaluated the effects of intraoperative ulinastatin on early-postoperative recovery in patients undergoing cardiac surgery. *Methods.* RCTs comparing intraoperative ulinastatin with placebo in cardiac surgery were searched through PubMed, Cochrane databases, Medline, SinoMed, and the China National Knowledge Infrastructure (1966 to May 20th, 2013). The primary endpoints included hospital mortality, postoperative complication rate, length of stay in intensive care unit, and extubation time. The physiological and biochemical parameters illustrating postoperative cardiac and pulmonary function as well as inflammation response were considered as secondary endpoints. *Results.* Fifteen RCTs (509 patients) met the inclusion criteria. Ulinastatin did not affect hospital mortality, postoperative complication rate, or ICU length of stay but reduced extubation time. Ulinastatin also increased the oxygenation index on postoperative day 1 and reduced the plasma level of cardiac troponin-I. Additionally, ulinastatin inhibited the increased level of tumor necrosis factor-alpha, polymorphonuclear neutrophil elastase, interleukin-6, and interleukin-8 associated with cardiac surgery.* Conclusion. *Ulinastatin may be of value for the inhibition of postoperative increased inflammatory agents and most likely provided pulmonary protective effects in cardiac surgery. However, larger adequately powered RCTs are required to define the clinical effect of ulinastatin on postoperative outcomes in cardiac surgery.

## 1. Introduction

The use of a cardiopulmonary bypass (CPB) during cardiac surgery leads to leukocyte (including neutrophil) activation, triggering a systemic inflammatory response [1], and inducing postoperative complications which include myocardial dysfunction [2, 3], acute lung injury [4], and multiorgan failure [5]. This contributes to prolonged postoperative recovery, delayed extubation time, and an extended intensive care unit (ICU) stay [6, 7].

Ulinastatin, one of the Kunitz-type human protease inhibitors found in urine, has the capacity to inhibit the neutrophilic elastase and the activation of proinflammatory cytokines [8], where it is anticipated that it could suppress the systematic inflammatory response associated with cardiac surgery. Although ulinastatin has been tested in many small sample sized clinical trials in cardiac surgery since the 1990s, where it has demonstrated the clear inhibitory effects in attenuating the postoperative increase in proinflammatory cytokines, its impact on clinical outcomes and postoperative complications remains controversial [9–15]. As a consequence, ulinastatin is not currently part of routine treatment for patients undergoing cardiac surgery [16]. We therefore undertook a systematic review and meta-analysis of randomized controlled trials (RCTs) which analyzed the effects of intraoperative ulinastatin treatment on postoperative clinical outcomes in patients undergoing cardiac surgery.

## 2. Materials and Methods

### 2.1. Literature Search, Inclusion, and Exclusion Criteria

We adopted the methods detailed in the Cochrane Handbook for Systematic Reviews version [17] and the guidelines of the PRISMA statement [18] for conduct of the meta-analysis. The search included the Cochrane Databases, Pubmed, Medline, SinoMed, and the China National Knowledge Infrastructure (CNKI) from 1966 to May 20th 2013, using the text words and medical subject headings (MeSH) comprising “ulinastatin” and “cardiac surgery”. The Cochrane Collaboration's highly sensitive search strategy (HSSS) was used to identify the relevant articles in the Cochrane Databases and Medline [19]. Text words and MeSH without HSSS were used to identify the relevant articles in Pubmed, SinoMed, and CNKI. ClinicalTrials.gov was also examined for relevant studies. Two reviewers (HQL and SHH) independently screened the titles and abstracts in order to select trials involving patients undergoing elective cardiac surgery who received intraoperative ulinastatin. Following the preliminary search, 253 articles were identified as potentially eligible and suitable for in-depth analysis.

Eligible inclusion criteria for the studies were that (1) prospective RCTs compare the ulinastatin group with the placebo group in cardiac surgery; (2) the baseline characteristics including age, sex, and type of operation were comparable between the groups; (3) the language of published studies was restricted to English, Chinese, and Japanese; (4) the period of follow-up was at least 24 hours after surgery. Exclusion criteria were that (1) the method of randomization was incorrect; (2) there were duplicate publications; (3) there was a lack of any data regarding the clinical endpoints; (4) studies were performed on a cohort of patients previously used for another trial. The full texts of highly relevant articles were thoroughly read and analyzed by the two reviewers.

### 2.2. Data Extraction and Outcome Measures

Descriptive data (e.g., patient population, type of surgery, intervention, and exclusion criteria) and markers of validity (e.g., methods of randomization, blinding) from all trials were extracted. Primary endpoints of interest were the hospital mortality, the early-postoperative complication rate, the length of stay in ICU, and extubation time. Secondary endpoints included the oxygenation index (OI), the cardiac index (CI) and the plasma levels of cardiac troponin-I (cTnI), creatine kinase MB isoenzyme (CK-MB), tumor necrosis factor-alpha (TNF-*α*), polymorphonuclear neutrophil elastase (PMNE), interleukin-6 (IL-6), and interleukin-8 (IL-8) on the postoperative first day (POD1). We accepted the authors' definitions for clinical outcomes. An early-postoperative complication was defined as organ dysfunction or an infective complication during the hospital stay as well as excessive bleeding requiring reoperation. The extubation time was defined as the duration (in hours) of postoperative mechanical ventilation. Two independent investigators (HQL and YF) abstracted the relevant data and measured the outcomes. When a discrepancy occurred, a third reviewer (SHH) acted as a referee so that a final consensus decision could be made.

### 2.3. Quality Scoring and Risk of Bias Assessment

The Jadad score was used for the quality assessment of the included trials [20]. Details of the quality assessment included the methodology of randomization, the adequacy of allocation concealment, whether a blind or double-blind method was used, whether an intention-to-treat analysis was utilized and descriptions of withdrawals, and follow-up. A risk of bias assessment was performed in accordance with guidelines outlined in the Cochrane handbook for Systematic Reviews or Interventions v.5.1.0. Two authors (HQL and ZF) reviewed all studies and subjectively assigned a value of “high”, “low”, or “unclear” to the following: (1) selection bias (was the randomization sequence adequate? was allocation concealment satisfactory?); (2) performance and detection bias (were participants, personnel, and outcome assessors blinded?); (3) attrition bias (were incomplete outcome data sufficiently assessed and dealt with?); (4) publication bias (was there evidence of selective outcome reporting?); and (5) any other sources of identifiable bias. The two trained reviewers (HQL and ZF) assessed the quality of the trials and the risk of bias independently resolving differences by consensus.

### 2.4. Statistical Analysis

An intention-to-treat analysis was used in the meta-analysis. The mean difference (MD) or odds ratios (ORs) and 95% confidence intervals (95% CIs) were calculated with the methods recommended by the Cochrane Collaboration [17]. The Cochrane's *χ* ^2^ test was used to detect heterogeneity among the studies. If the *P* value of Cochrane's *χ* ^2^ test was more than 0.05, a fixed effects model was employed and the Mantel-Haenszel method was performed to analyze the data. If the *P* value was less than 0.05, heterogeneity was explored. Egger's regression was used to detect publication bias [21]. Meta-analysis was conducted using Review manager 5 (version 5.0.14; Copenhagen: the Nordic Cochrane Centre, the Cochrane Collaboration) and Egger's regression was performed with the Stata 10.0 software (Stata Corp, USA).

## 3. Results

### 3.1. Characteristics of Included Studies

**Figure 1:**
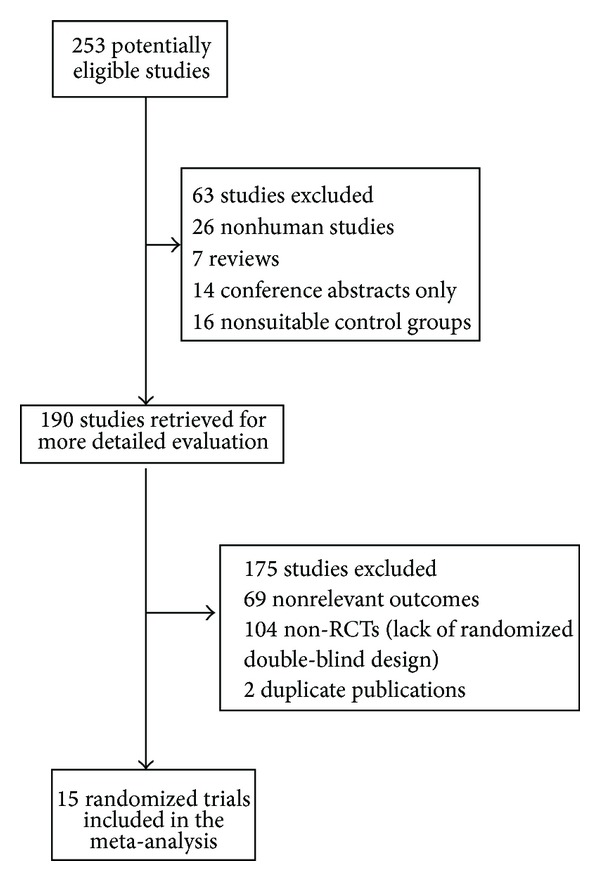
Process of trial selection.

**Figure 2:**
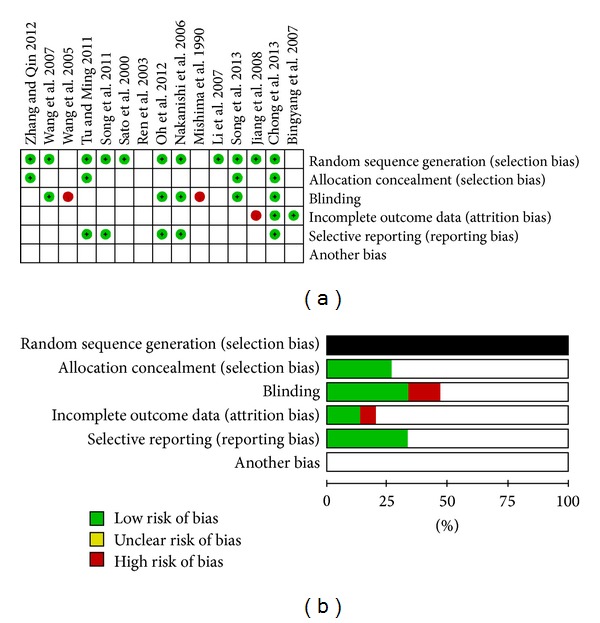
Risk of bias summary.

Fifteen studies involving 509 participants were included in the meta-analysis [22–36].  Figure 1 depicts the flow chart of the selection process. The characteristics of included studies are shown in Table 1. None of the studies was a multicenter RCT. The median sample size of the RCTs was 30 patients (range 15–60). With regard to the types of surgery, one trial assessed only aortic arch replacements [36], four trials only evaluated coronary artery bypass grafts (CABG) [23, 25, 29, 30], five trials reported only valve surgery [26, 28, 33–35], three trials evaluated only repairs of atrial or ventricular septal defects [22, 24, 32], and two trails were mixed in nature [27, 31]. All trials except one [25] were conducted with CPB. Ulinastatin treatment protocols varied in their dosage and administration times. The risk of bias analysis (Figure 2) showed that eleven studies described their randomization methods [22, 25, 27–29, 31–36] and that five studies were double-blind [25, 28, 29, 35, 36]. Because all included studies were RCTs, the Jadad score for all studies was >2 with a mean overall Jadad score of 3.6 ± 1.1. Table 1 shows that twelve of the studies with a Jadad score > 3 were considered high-quality RCTs [20] and that four studies had a Jadad score = 5 [28, 29, 33, 35].

### 3.2. Data Synthesis

Table 2 summarizes the results of the meta-analysis for each outcome.

### 3.3. Hospital Mortality

**Figure 3:**
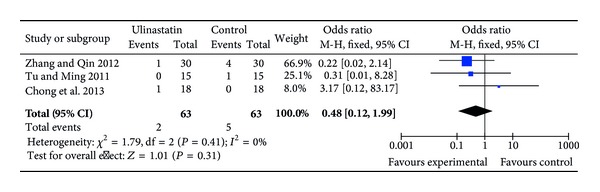
Impact of ulinastatin on hospital mortality. M-H = Mantel-Haenszel, 95% CI = 95% confidence intervals, Chi^2^= Chi-square test, df = degrees of freedom,* I* ^2^ =* I* ^2^ index (quantify the degree of heterogeneity), and* Z* =* Z* test.

Nine RCTs reported data on hospital mortality [22, 24, 26, 27, 29, 30, 32, 34–36]. There were a total of 7 postoperative deaths (7/306, 2.3%). Two patients in the ulinastatin group died of coagulapathy [36] and cardiac infraction [27], respectively. Myocardial infarction and respiratory failure were the main causes of death in the control group. Since 6 of these trials did not present any hospital deaths, where all of the participants survived (“zero-sum” studies), the remaining 3 RCTs (126 patients) [27, 32, 36] were used to perform the meta-analysis. Ulinastatin treatment did not influence overall hospital mortality (OR = 0.48; 95% CI, 0.12 to 1.99; *P* = 0.31; Cochrane's *χ* ^2^ test, *P* = 0.41) (Figure 3).

### 3.4. Early-Postoperative Complication Rates

**Figure 4:**
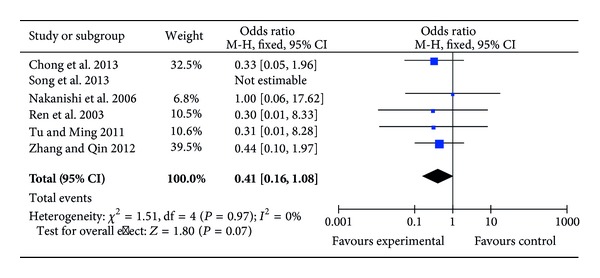
Impact of ulinastatin on early-complication rate. M-H = Mantel-Haenszel, 95% CI = 95% confidence intervals, Chi^2^= Chi-square test, df = degrees of freedom,* I* ^2^ =* I* ^2^ index, and* Z* =* Z* test.

Twelve RCTs reported data on early-postoperative complications [22, 24, 26, 27, 29–36] with 6 trials being zero-sum studies which reported that there were no patient suffering postoperative complications due to operation or CPB procedures [22, 26, 30, 31, 33, 34]. In the ulinastatin groups of the remaining 6 RCTs, postoperative complications included 6 cases of myocardial ischemia [27, 29, 35], 1 wound infection [36], 1 reoperation for bleeding [36], 7 respiratory failures [35], and 4 renal failures [35], whilst in the control groups, there were 13 cases of myocardial ischemia [27, 29, 36], 3 patients with excessive bleeding [24, 36], 4 respiratory failures [32, 35], 1 wound infection [36], and 5 renal failures [35, 36] (Table 3). As only one trial [35] reported the number of comorbidities, which was more than the total number of patients, the remaining 5 RCTs (176 patients) [24, 27, 29, 32, 36] were estimable to perform the analysis showing that ulinastatin treatment did not influence early-postoperative complication rate (OR = 0.41; 95% CI, 0.16 to 1.08; *P* = 0.07; Cochrane's *χ* ^2^ test, *P* = 0.97) (Figure 4).

### 3.5. ICU Length of Stay

**Figure 5:**
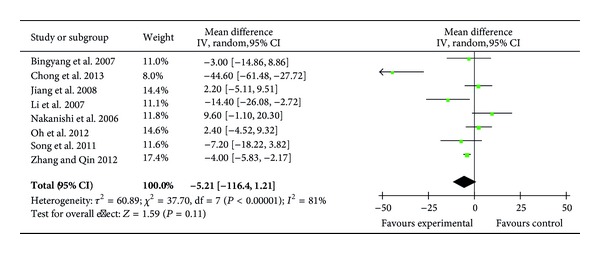
Impact of ulinastatin on length of stay in ICU (hours). ICU = intensive care unit, IV = inverse variance, 95% CI = 95% confidence intervals, Tau^2^ = Tau-squared test, Chi^2^ = Chi-square test, df = degrees of freedom,* I* ^2^ =* I* ^2^ index, and* Z* =* Z* test.

Eight RCTs (318 patients) evaluated the effect of ulinastatin on the length of stay in ICU [22, 27–30, 33, 34, 36]. Four of these trials reported a significantly shorter ICU length of stay in the ulinastatin treatment groups [27, 33, 34, 36]; however, meta-analysis using the random effects model showed that the ulinastatin treatment did not significantly decrease the length of stay in ICU (MD = −5.21 h; 95% CI, −11.64 h to 1.21 h; *P* = 0.11; Cochrane's *χ* ^2^ test, *P* < 0.00001) (Figure 5).

### 3.6. Extubation Time

**Figure 6:**
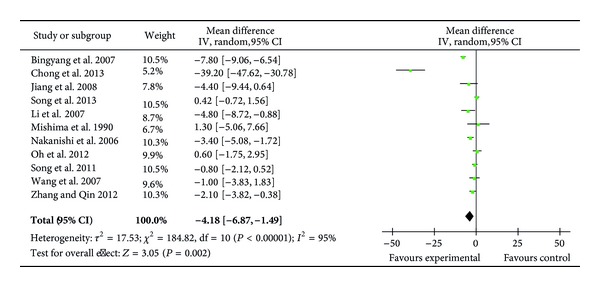
Impact of ulinastatin on extubation time (hours). IV = inverse variance, 95% CI = 95% confidence intervals, Tau^2^ = Tau-squared test, Chi^2^ = Chi-square test, df = degrees of freedom,* I* ^2^ =* I* ^2^ index, and* Z* =* Z* test.

Eleven RCTs (385 patients) reported extubation time [22, 23, 25, 27–30, 33–36] with 5 of the trials recording a significantly reduced extubation time in the ulinastatin groups (27, 29, 30, 34, 36). Meta-analysis showed that ulinastatin treatment significantly reduced extubation time (MD = −4.18 h; 95% CI, −6.87 h to −1.49 h; *P* = 0.002; Cochrane's *χ* ^2^ test, *P* < 0.00001) (Figure 6). Furthermore, to investigate the impact of total ulinastatin dosage on the outcome, we undertook an exploratory subgroup analysis and found that trials with a total dosage < 10,000 U/kg (206 patients) [23, 25, 27, 29, 33, 35] had a negative outcome (*P* = 0.08), whereas those with total dosage > 10,000 U/kg had a positive outcome (MD = −9.86 h; 95% CI, −16.58 h to −3.14 h, *P* = 0.004). Similar subgroup analysis of the isolated CABG trials (104 patients) [23, 25, 29, 30] and isolated valve repair trials (156 patients) [28, 33–35] showed no difference between ulinastatin-treated and control groups (*P* = 0.08 and *P* = 0.50, resp.).

### 3.7. Postoperative OI and CI

**Figure 7:**
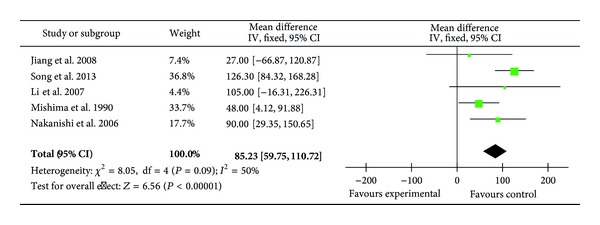
Impact of ulinastatin on postoperative oxygenation index (OI); IV = inverse variance, 95% CI = 95% confidence intervals, Chi^2^ = Chi-square test, df = degrees of freedom,* I* ^2^ =* I* ^2^ index, and* Z* =* Z* test.

Five trials (127 patients) reported the postoperative oxygenation indexes (OI) [22, 23, 29, 34, 35] which was increased by ulinastatin treatment (MD = 85.23; 95% CI, 59.75 to 110.72; *P* < 0.00001; Cochrane's *χ* ^2^ test, *P* = 0.09) (Figure 7).

**Figure 8:**
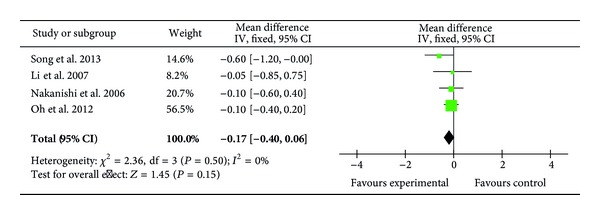
Impact of ulinastatin on postoperative cardiac index (CI). IV = inverse variance, 95% CI = 95% confidence intervals, Chi^2^ = Chi-square test, df = degrees of freedom,* I* ^2^ =* I* ^2^ index, and* Z* =* Z* test.

Four trials (138 patients) reported the postoperative cardiac indexes (CI) [28, 29, 34] showing no difference between the ulinastatin-treated and the control groups (MD = −0.17; 95% CI, −0.40 to 0.06; *P* = 0.15; Cochrane's *χ* ^2^ test, *P* = 1.00) (Figure 8).

### 3.8. Myocardial Damage Agents (cTnI and CKMB)

**Figure 9:**
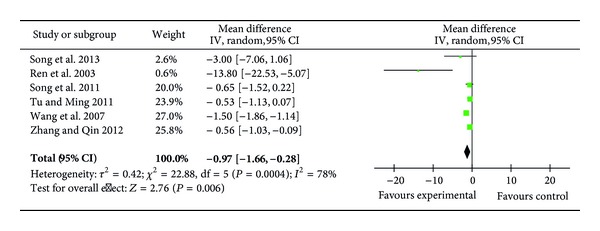
Impact of ulinastatin on the level of cTnI on postoperative first day (POD1). cTnI = cardiac troponin-I, IV = inverse variance, 95% CI = 95% confidence intervals, Tau^2^ = Tau-squared test, Chi^2^ = Chi-square test, df = degrees of freedom,* I* ^2^ =* I* ^2^ index, and* Z* =* Z* test.

Six trials (206 patients) reported the levels of plasma cTnI on postoperative first day (POD1) [24, 25, 27, 32, 33, 35] and showed that ulinastatin treatment significantly reduced the level of cTnI (MD = −0.97 ng/mL; 95% CI, −1.66 ng/mL to −0.28 ng/mL; *P* = 0.006; Cochrane's *χ* ^2^ test, *P* = 0.0004) (Figure 9).

**Figure 10:**
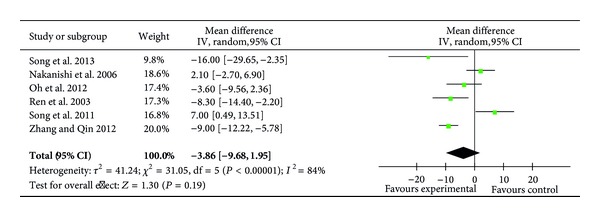
Impact of ulinastatin on the level of CKMB on postoperative first day (POD1). CKMB = creatine kinase MB isoenzyme, IV = inverse variance, 95% CI = 95% confidence intervals, Tau^2^ = Tau-squared test, Chi^2^ = Chi-square test, df = degrees of freedom,* I* ^2^ =* I* ^2^ index, and* Z* =* Z* test.

Six trials (242 patients) reported the levels of plasma CKMB on POD1 [24, 27–29, 33, 35] with recording a significantly higher CKMB level in the ulinastatin group [33], whereas 3 trials reported significantly lower CKMB levels following ulinastatin therapy [24, 27, 35]. Overall, meta-analysis showed no difference between ulinastatin groups and control groups (MD = −3.86 ng/mL; 95% CI, −9.68 ng/mL to 1.95 ng/mL;*P* = 0.19; Cochrane's *χ* ^2^ test, *P* < 0.00001) (Figure 10).

### 3.9. Plasma Inflammatory Agents (TNF-*α*, PMNE, IL-6, and IL-8)

**Figure 11:**
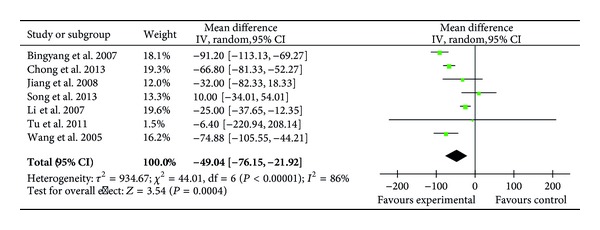
Impact of ulinastatin on the level of TNF-*α* on postoperative first day (POD1). TNF-*α* = tumor necrosis factor-alpha, IV = inverse variance, 95% CI = 95% confidence intervals, Tau^2^ = Tau-squared test, Chi^2^ = Chi-square test, df = degrees of freedom,* I* ^2^ =* I* ^2^ index, and* Z* =* Z* test.

Seven RCTs (203 patients) reported the levels of plasma TNF-*α* on POD1 [22, 26, 30, 32, 34–36]. Meta-analysis showed that ulinastatin treatment significantly inhibited the increased level of TNF-*α* (MD = −49.04 pg/mL; 95% CI, −76.15 pg/mL to −21.92 pg/mL; *P* = 0.0004; Cochrane's *χ* ^2^ test, *P* < 0.00001) (Figure 11).

**Figure 12:**
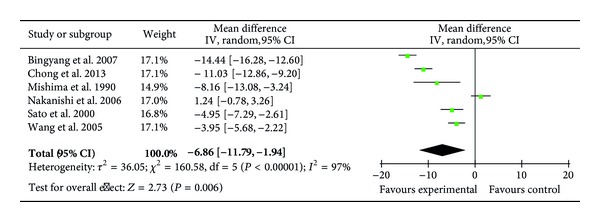
Impact of ulinastatin on the level of PMNE on postoperative first day (POD1). PMNE = polymorphonuclear neutrophil elastase, IV = inverse variance, 95% CI = 95% confidence intervals, Tau^2^ = Tau-squared test, Chi^2^ = Chi-square test, df = degrees of freedom,* I* ^2^ =* I* ^2^ index, and* Z* =* Z* test.

Six RCTs (161 patients) reported the levels of plasma PMNE on POD1 [23, 26, 29–31, 36] showing a decrease on POD 1 after ulinastatin treatment (MD = −6.86 *μ*g/dL; 95% CI, −11.79 *μ*g/dL to −1.94 *μ*g/dL; *P* = 0.006) (Figure 12). As 4 of these studies focused on CABG, an exploratory subgroup analysis was performed and showed no difference between the ulinastatin-treated and the control groups (MD = −6.55 *μ*g/dL; 95% CI, −14.46 *μ*g/dL to 1.35 *μ*g/dL; *P* = 0.10).

**Figure 13:**
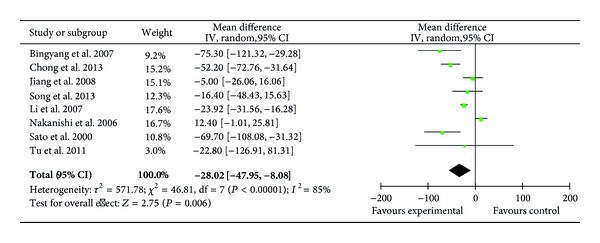
Impact of ulinastatin on the level of IL-6 on postoperative first day (POD1). IL-6 = interleukin-6, IV = inverse variance, 95% CI = 95% confidence intervals, Tau^2^ = Tau-squared test, Chi^2^ = Chi-square test, df = degrees of freedom,* I* ^2^ =* I* ^2^ index, and* Z* =* Z* test.

Eight RCTs (219 patients) reported the levels of plasma IL-6 on POD1 [22, 29–32, 34–36] where ulinastatin significantly decreased the level of IL-6 on POD 1 (MD = −28.02 pg/mL; 95% CI, −47.95 pg/mL to −8.08 pg/mL; *P* = 0.006) (Figure 13). But when analyzing the CABG surgery subgroups (75 patients) [29–31], ulinastatin had no effect (MD = −41.78 pg/mL; 95% CI, −108.25 pg/mL to −24.68 pg/mL; *P* = 0.22).

**Figure 14:**
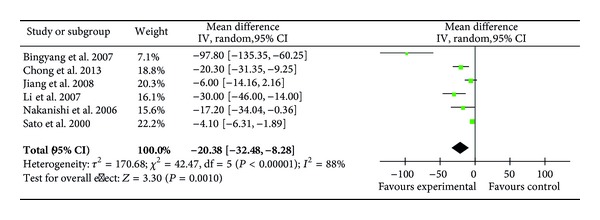
Impact of ulinastatin on the level of IL-8 on postoperative first day (POD1). IL-8 = interleukin-8, IV = inverse variance, 95% CI = 95% confidence intervals, Tau^2^ =Tau-squared test, Chi^2^ = Chi-square test, df = degrees of freedom,* I* ^2^ =* I* ^2^ index, and* Z* =* Z* test.

Six RCTs (165 patients) reported the levels of plasma IL-8 on POD1 [22, 29–31, 34] showing a significantly decrease with ulinastatin treatment (MD = −20.38 pg/mL; 95% CI, −32.48 pg/mL to −8.28 pg/mL; *P* = 0.001) (Figure 14).

### 3.10. Sensitivity Analysis for Study Quality

We undertook subgroup meta-analysis including exclusively high-quality studies (Jadad score ≥ 3) in order to find how the quality of studies influenced the outcomes of our meta-analysis. Regarding the clinical and physiologic outcomes, our conclusion among high-quality RCTs remained consistent. But for biologic outcomes, the meta-analysis of 5 high-quality studies [22, 33–36] indicated that ulinastatin did not have inhibitory effect on plasma levels of TNF-*α* (*P* = 0.08); and meta-analysis of 3 high-quality studies [29, 31, 36] showed no difference in plasma levels of PMNE between the ulinastatin-treated and the control groups either (*P* = 0.20).

## 4. Discussion

Our meta-analysis showed a significant decrease in the extubation time for ulinastatin-treated patients but no effect on hospital mortality, early postoperative complication rate, or ICU length of stay when compared with controls. In an assessment of secondary outcome measures, ulinastatin increased the oxygenation index (OI) but not the cardiac index (CI) and reduced the plasma levels of cTnI but not CK-MB measured on the first postoperative day. Additionally, ulinastatin significantly inhibited the increased postoperative level of PMNE, TNF-*α*, IL-6, and IL-8 associated with cardiac surgery.

The decrease in extubation time and the increased OI indicate that the intraoperative use of ulinastatin does provide some clinical benefits such as the improvement of early postoperative pulmonary function, a finding which has been reported previously [10, 23, 37–39].

Cardiopulmonary bypass (CPB) and cardiac surgery usually induce an activation and release of neutrophil and proinflammatory cytokines [40], most notably IL-6 and IL-8, which may be early prognosis factors for organ dysfunction following cardiac surgery [41]. In this respect, it was reported that the removal by ultrafiltration of inflammatory substances from the circulation including inflammatory cytokines and scavenger toxins may improve early postoperative organ function after cardiac surgery [2, 42].

The transcription of IL-6, IL-8, and TNF-*α* is a secondary event induced by the bioactive IL-1 beta [43, 44], where it is anticipated that the protease inhibitor, ulinastatin which has inhibitory effects on neutrophilic elastase and on the conversion of prointerleukin 1 beta (pro-IL-1; 31 kDa peptide, inactive) to IL-1 beta (17 kDa peptide, active) [43, 45], will attenuate the acute phase response. Consistent with these results in animals [43–45] as well as in patients undergoing major operations (such as hepatectomy) or in those with mutitrauma [46–55], the finding of the present meta-analysis focusing on cardiac surgery would suggest that intraoperative ulinastatin inhibits the sequestration and activation of neutrophil and attenuates the normal postoperative rise in cytokines, reducing the systemic inflammatory response syndrome, pulmonary microvascular permeability, and postoperative lung edema [55–57]. These findings would correlate with a higher OI in ulinastatin-treated cases and a more rapid extubation time consequent upon a more aggressive policy by intensive care physicians to more rapidly wean their patients from mechanical ventilation. Moreover, a decreased extubation time may protect patients from ventilator-associated pneumonia and contribute to a reduction in the length of ICU stay and the hospital cost along with a diminution in psychosocial and physical risks to the patient and even death [58, 59]. Interestingly, in our study, subgroup analysis concerning the total dose of ulinastatin indicates that a higher dosage protocol significantly reduced extubation time which may suggest a dose-dependent effect of ulinastatin on pulmonary protection. High-quality RCTs comparing different dosages of ulinastatin are required in order to answer this question.

The reduced periods of intubation with ulinastatin impact on the length of ICU stay where 4 trials included in the analysis confirmed a lesser length of ICU stay with ulinastatin therapy [27, 33, 34, 36] (Figure 4), although meta-analysis on all 8 trials which reported the length of ICU stay did not show a significant effect. Concerning this point, the factors affecting the length of ICU stay in patients undergoing cardiac surgery are ambiguous, including the basic preoperative cardiac function of patient, the length of the CPB, the recovery of major organ function, new onset atrial fibrillation, a stuck mechanical valve, the presence of postoperative bleeding, and unknown iatrogenic factors [60–62]. It is expected that these factors should be equally distributed within the RCTs examined for this meta-analysis. It is further likely that conflicting reports on the postoperative effects of ulinastatin will affect the meta-analysis of ICU stay, reflecting the heterogeneity of RCTs, some of which did not define standards of ICU discharge [22, 33] or report on postoperative complications [29, 35].

Ulinastatin also significantly decreased the postoperative levels of cTnI. Normally, it has been shown that surgical procedures as well as CPB during cardiac surgery induce a systemic acute inflammatory response and regional myocardial I/R injury leading to increased endothelial permeability and free radical damage to vessels and parenchyma with coincident myocardial damage [63]. Both cTnI and CK-MB are predictive factors which reflect myocardial injury where cTnI is the most sensitive indicator of minor myocardial damage with superior cardiac specificity when compared with CK-MB [64–66]. The decreased levels of cTnI would suggest a potential role for ulinastatin as a myocardial protective agent, although the observed clinical outcomes, (e.g. incidence of postoperative myocardial ischemia) could not be determined. Studies involving larger patient numbers are required; however, it may, well, be that there are more specific clinical outcome indicators worth assessing such as the occurrence of delayed myocardial ischemia up to 30 or 60 days after surgery.

Recently, an increasing body of evidence has highlighted the role of ulinastatin in postoperative mortality and morbidities with controversial results [22, 24, 26, 27, 29, 30, 32, 34, 35, 50, 52, 54, 67–69]. Interpretation of these trials is difficult since there is considerable heterogeneity, particularly regarding the type of surgery, the length of CPB, and the dosage of ulinastatin used. Further, the majority of the trials focused on low-risk patients, which may result in a much lower observed death rate (2.3%) than would normally be expected with CPB alone (3.2% to 12.8%) [70]. Without clear definitions, tracking complication events such as myocardial infarction after cardiac surgery is likely to be underreported, limiting the value of the meta-analytic approach in the assessment of both hospital mortality and postoperative complication rates. Although our analysis showed a trend of favoring a decreased complication rate with ulinastatin, larger, adequately powered and well-designed RCTs are required to better elucidate the impact of perioperative ulinastatin.

Despite the rigorous nature of the analysis and a high agreement between observers, our study has several limitations. Firstly, the majority of studies focused on biochemical markers of inflammation with relatively poor descriptions of the secondary clinical outcomes, an effect likely to result in significant underreporting of perioperative adverse events. Secondly, the small sample size in many cases makes interpretation guarded where it cannot be assumed that variables like ICU stay and extubation times are normally distributed. Thirdly, the diversity of the surgeries performed, the ulinastatin doses utilized, and the timing of ulinastatin administration (intra-* versus *postoperative) [32, 34] are confounding factors. The analysis of studies over a 20-year period where there have been considerable changes in cardiac surgery and anesthesia as well as in CPB technology, myocardial protection, and antifibrinolytic therapies will also influence the overall results.

In conclusion, it remains insufficient evidence to support a beneficial effect of ulinastatin on mortality, the postoperative complication rate, or the length of ICU stay following cardiac surgery. Analysis shows, however, that intraoperative ulinastatin might provide protective effects on cardiac and pulmonary function, reducing plasma levels of cTnI, increasing the oxygenation index, and reducing the extubation time. These effects might be associated with a concomitant inhibition of neutrophilic elastase and an attenuation of the normal rise in proinflammatory cytokines normally detected on the first postoperative day after cardiac surgery.

## Figures and Tables

**Table 1:** Characteristics of included trials.

Studies	*n*	Mean age	Type of surgery	CPB time (min)		Intervention	Primary endpoints	Jadad Score		
				Ulinastatin	Control			Randomization	Blinding	Withdrawals
Mishima et al., 1990 [23]	20	57.8	CABG	155	163	Grp1: 5000 U/kg i.v. drip before CPB (*n* = 10) Grp 2: Placebo (*n* = 10)	ET, OI, PMNE	1	0	1

Sato et al., 2000 [31]	15	65	CABG, repair of VSD, ASD	155	191	Grp1: 600,000 U into priming solution, 300,000 U i.v. drip before removal of aortic clamping, 150,000 U i.v. drip Bid for 5 days (*n* = 7) Grp 2: blank (*n* = 8)	Complication rate, IL-6, IL-8, PMNE	2	1	1

Ren et al., 2003 [24]	20	6.7	repair of VSD, ASD	Not stated		Grp1: 6000 U/kg into priming fluid and 6000 U/kg i.v. before aorta cannulation (*n* = 10) Grp2: Placebo (*n* = 10)	Complication rate, cTnI, CKMB	1	1	1

Wang et al., 2005 [26]	30	35.9	valve surgery	68.2	82.1	Grp1: 10,000 U/kg into primary solution and 10,000 U/kg i.v. before CPB (*n* = 15) Grp2: Placebo (*n* = 15)	Complication rate, OI, TNF-*α*, PMNE	1	0	1

Nakanishi et al., 2006 [29]	30	62	CABG	150	135	Grp1: 5000 U/kg i.v. before aortic cannulation (*n* = 15) Grp 2: Placebo (*n* = 15)	Complication rate, ICU stay, OI, CI, ET, cTnI, CKMB, IL-6, IL-8	2	2	1

Bingyang et al., 2007 [30]	30	57.4	CABG	108	117	Grp1: 300,000 U i.v. before CPB, 300,000 U i.v. before aortic clamping release, 400,000 U i.v. after protamine (*n* = 15) Grp 2: Placebo (*n* = 15)	Complication rate, ICU stay, ET, PMNE, IL-8, TNF-*α*, IL-6	1	0	1

Wang et al., 2007 [25]	24	57	CABG	No CPB		Grp 1: 6000 U/kg i.v. after induction of anesthesia and 1000 U·kg^−1^·h^−1^ i.v. to the end of operation (*n* = 12) Grp 2: Placebo (*n* = 12)	ET, cTnI	2	2	0

Li et al., 2007 [34]	24	52.5	valve surgery	91.3	97.2	Grp 1: 50,000 U/kg i.v. Q 12 h for postop. 3 days (*n* = 12) Grp 2: Placebo (*n* = 12)	ICU stay, ET, OI, CI, IL-6, IL-8, TNF-*α*	2	0	1

Jiang et al., 2008 [22]	58	3.3	repair of VSD and ASD	52	58	Grp 1: 20,000 U/kg into the prime solution (*n* = 15) Grp 2: Placebo (*n* = 14)	ICU stay, ET, OI, IL-6, IL-8, TNF-*α*	2	0	1

Song et al., 2011 [33]	48	54	valve surgery	170	172	Grp1: 5000 U/kg i.v. before aortic cross clamping (*n* = 24) Grp 2: Placebo (*n* = 24)	Complication rate, ICU stay, ET, cTnI, CKMB	2	2	1

Tu and Ming 2011 [32]	30	3.6	repair of VSD and ASD	76	70	Grp1: 5000 U/kg i.v. drip Tid for 3 days (*n* = 15) Grp2: blank (*n* = 15)	Hospital mortality, complication rate, IL-6, TNF-*α*, cTnI	2	0	1

Zhang and Qin 2012 [27]	60	62.8	CABG, valve surgery	98	95	Grp1: 150,000 U i.v. drip after induction of anesthesia and 150,000 U i.v. drip after CPB (*n* = 30) Grp 2: Placebo (*n* = 30)	Hospital mortality, complication rate, ICU stay, ET, CKMB, cTnI	2	1	1

Oh et al., 2012 [28]	60	67	valve surgery	99	96	Grp1: 300,000 U i.v. drip after induction of anesthesia, 400,000 U into CPB prime solution, and 300,000 U after weaning from CPB (*n* = 30) Grp 2: Placebo (*n* = 30)	ICU stay, ET, CKMB, CI	2	2	1

Chong et al., 2013 [36]	36	54.8	aortic arch replacement	235.5	247.2	Grp1: 20,000 U/kg i.v. drip in total: 1/3 after anesthesia induction, 1/3 before aortic cross-clamp, and 1/3 after aortic clamp release (*n* = 18) Grp 2: Placebo (*n* = 18)	Hospital mortality, complication rate, ICU stay, ET, IL-6, IL8, PMNE, TNF-*α*	2	2	0

Song et al., 2013 [35]	24	58	valve repair	150	139	Grp1: 5,000 U/kg i.v. drip after the opening of a pericardium (*n* = 13) Grp 2: Placebo (*n* = 11)	ET, OI, CI, cTnI, CKMB, IL-6, TNF-*α*	2	2	1

CABG: coronary artery bypass graft, VSD: ventricular septal defect, ASD: atrial septal defect, CPB: cardiopulmonary bypass, UTI: urinary trypsin inhibitor, Grp: group, postop.: postoperative, Bid: twice a day, Tid: three times a day, i.v.: intravenous, ET: extubation time, OI: oxygenation index, PMNE: polymorphonuclear neutrophil elastase, IL-6: interleukin-6, IL-8: interleukin-8, cTnI: cardiac troponin-I, CK-MB: creatine kinase MB isoenzyme, TNF-∝: tumor necrosis factor-alpha, CI: cardiac index.

**Table 2:** Summary of effects of ulinastatin treatment on postoperative outcomes.

Outcome	Number of studies	Total *N*	OR^#^/MD	95% CI	*P* value	*I* ^2^ (%)
Clinical						
Hospital mortality	3	126	0.48^#^	0.12 to 1.99	0.31	0
Complication rate	5	176	0.41^#^	0.16 to 1.08	0.07	0
ICU stay (h)	8	318	−5.21	−11.64 to 1.21	0.11	81
Extubation time (h)	11	385	−4.18	−6.87 to −1.49	0.002	95
Physiologic						
cTnI (ng/mL)	6	206	−0.97	−1.66 to −0.28	0.006	78
CKMB (ng/mL)	6	242	−3.86	−9.68 to 1.95	0.19	84
Postoperative OI	5	127	85.23	59.75 to 110.72	<0.00001	50
Postoperative CI	4	138	−0.10	−0.32 to 0.12	0.39	0
Biologic						
TNF-*α* (pg/mL)	7	203	−49.04	−76.15 to −21.92	0.0004	86
PMNE (*μ*g/dL)	6	161	−6.86	−11.79 to −1.94	0.006	97
IL-6 (pg/mL)	8	219	−28.02	−47.95 to −8.08	0.006	85
IL-8 (pg/mL)	6	129	−20.38	−32.48 to −8.28	0.001	88

ICU: intensive care unit, POMV: postoperative mechanic ventilation, TNF-*α*: tumor necrosis factor-alpha, PMNE: polymorphonuclear neutrophil elastase, IL-6: interleukin-6, IL-8: interleukin-8, cTnI: cardiac troponin-I, CK-MB: creatine kinase MB isoenzyme, OI: oxygenation index, CI: cardiac index, OR: odds ratios, MD: mean difference, 95% CI: 95% confidence intervals.

^#^OR: odds ratio.

**Table 3:** Incidence of early-postoperative complications for included trials.

Study	Group	Complications (cases)				
		Myocardial infarction	Wound infection	Excessive bleeding	Respiratory failure	Renal failure
Chong et al., 2013 [36]	U	0	1	1		0
	C	1	1	2		1
Nakanishi et al., 2006 [29]	U	1				
	C	1				
Ren et al., 2003 [24]	U			0		
	C			1		
Tu and Ming, 2011 [32]	U				0	
	C				1	
Zhang and Qin, 2012 [27]	U	3				
	C	6				
Song et al., 2013 [35]	U	2			7	4
	C	5			3	4

U: ulinastatin group, C: control group.
